# Frecuencia de algunas enfermedades que afectan la salud de hombres de Andes, Antioquia

**DOI:** 10.15446/rsap.V27n1.114072

**Published:** 2025-01-01

**Authors:** Girlesa Jaramillo-Muñoz, Walter D. Cardona-Maya, Jenniffer Puerta-Suárez

**Affiliations:** 1 GJ: Biol. Grupo Reproducción, Facultad de Medicina, Universidad de Antioquia. Medellín, Colombia. gruporeproduccion@udea.edu.co Universidad de Antioquia Facultad de Medicina Universidad de Antioquia Medellín Colombia gruporeproduccion@udea.edu.co; 2 WC: Bact. M. Sc. Ciencias Básicas Biomédicas. Ph. D. Biología. Grupo Reproducción, Departamento de Microbiología y Parasitología, Facultad de Medicina, Universidad de Antioquia. Medellín, Colombia. wdario.cardona@udea.edu.co Universidad de Antioquia Facultad de Medicina Universidad de Antioquia Medellín Colombia wdario.cardona@udea.edu.co; 3 JP: Microbiol. Bioan. M. Sc. Biología. Ph. D. Biología. Grupo Reproducción, Departamento de Obstetricia y Ginecología, Facultad de Medicina, Universidad de Antioquia. Medellín, Colombia. jenniffer.puerta@udea.edu.co Universidad de Antioquia Departamento de Obstetricia y Ginecología Facultad de Medicina Universidad de Antioquia Medellín Colombia jenniffer.puerta@udea.edu.co

**Keywords:** Salud, enfermedad, reproducción, fertilidad, hombres *(fuente: DeCS, BIREME)*, Health, disease, reproduction, fertility, men *(source: MeSH, NLM)*

## Abstract

**Objetivo:**

Describir la frecuencia de algunas enfermedades que afectan la salud de los hombres residentes en el municipio de Andes.

**Materiales y Métodos:**

Estudio cuantitativo, retrospectivo, en el que se describió y comparó la frecuencia de algunas enfermedades reportadas por el sistema de vigilancia en salud de la Secretaría de Salud de Andes y la Secretaría Seccional de Salud y Protección Social de Antioquia.

**Resultados:**

Se observó un posible subregistro de los datos epidemiológicos reportados en el sistema de vigilancia en salud, al comparar los resultados entregados por la autoridad local con aquellos proporcionados por la autoridad departamental, lo que dificultó obtener resultados contundentes. Sin embargo, en general, las principales enfermedades que afectan la salud de los hombres de Andes son, en orden de frecuencia, la diabetes, la obesidad, el alcoholismo, la violencia intrafamiliar, el cáncer de próstata, la infección por el virus de la inmunodeficiencia humana y la infección por *Neisseria gonorrhoeae.*

**Discusión:**

En Colombia, la mayoría de los servicios en salud sexual están enfocados en las mujeres, y el hombre representa un papel secundario. Enfermedades como la diabetes, la obesidad y el alcoholismo, así como la violencia intrafamiliar, los tumores malignos de la próstata, el VIH y la infección por *N. gonorrhoeae* afectan la salud masculina, pero también pueden tener impacto en la salud sexual.

**Conclusión:**

La diabetes y la obesidad son los principales problemas de salud que afectan a los hombres de Andes y también pueden afectar su salud sexual, por lo que se requieren estrategias en salud especiales para la atención de los hombres.

## Abstract (en)

**Objective:**

To describe the frequency of some diseases that affect the health of men living in the municipality of Andes.

**Materials and Methods:**

A quantitative, retrospective study was conducted in which the frequency of some diseases reported by the health surveillance system of the Ministry of Health of Andes and the Sectional Secretariat of Health and Social Protection of Antioquia was described and compared.

**Results:**

A possible underreporting of the epidemiological data reported in the health surveillance system was observed, when comparing the results provided by the local authority with those provided by the departmental authority, which made it difficult to obtain conclusive results. However, in general, the main diseases that affect the health of men in Andes are, in order of frequency, diabetes, obesity, alcoholism, domestic violence, prostate cancer, human immunodeficiency virus infection, and *Neisseria gonorrhoeae* infection.

**Discussion:**

In Colombia, most sexual health services are focused on women, and men play a secondary role. Diseases such as diabetes, obesity and alcoholism, as well as domestic violence, malignant prostate tumors, HIV and *N. gonorrhoeae* infection affect men's health, but can also have an impact on sexual health.

**Conclusion:**

Diabetes and obesity are the main health problems that affect men in Andes and can also affect their sexual health, so special health strategies are required for men's care.

La salud sexual y reproductiva (SSR) comprende el bienestar físico, mental y social de las personas y no solo la ausencia de enfermedades en lo relacionado con la sexualidad 1, esta procura defender y brindar calidad a la sexualidad y a las relaciones interpersonales e incorporar algunas características socioculturales que asignan funciones específicas a hombres y a mujeres, mientras que la salud reproductiva comprende los métodos, las técnicas y los servicios para abordar los problemas reproductivos. En Colombia, el Ministerio de Salud y Protección Social ha implementado políticas para garantizar los derechos en SSR, aunque generalmente centralizadas y aplicadas con mayor frecuencia en las grandes ciudades del territorio colombiano; en cambio, en municipios, corregimientos o veredas se tiene menor participación, asociado principalmente a su aislamiento geográfico y al abandono de las poblaciones pequeñas por parte de los gobiernos.

Las políticas públicas en materia de SSR suelen enfocarse en los problemas que afectan a las mujeres, quienes finalmente, en países donde es muy frecuente la conducta machista, como es el caso de Colombia, llevan solas el proceso de embarazo, parto y puerperio, por lo que se desconoce el estado de SSR de los hombres, hasta el punto de que estos ignoren algunos conceptos básicos de este tema y solo se interesen en momentos específicos como cuando son diagnosticados con algunas infecciones de transmisión sexual o presentan dificultades para embarazar a sus parejas 2. Adicionalmente, en las áreas rurales los servicios en SSR son escasos, debido al incremento en los casos por las dificultades en el acceso geográfico. Lo anterior restringe los servicios a políticas de planificación familiar en mujeres y al cumplimiento del programa ampliado de inmunización, además de afectar la inclusión del género masculino. Como un agravante de la situación descrita, son pocos los estudios sobre SSR enfocados en el género masculino, factor que limita la inclusión y la diversidad cultural 2. Teniendo en cuenta las limitaciones previamente descritas, el objetivo del presente trabajo es describir la frecuencia de algunas enfermedades que afectan la salud de los hombres residentes en el municipio de Andes. Para cumplir con este objetivo se hizo uso de la información epidemiológica de los sistemas de vigilancia en salud departamentales y locales.

## MATERIALES Y MÉTODOS

Estudio cuantitativo, retrospectivo, en el que se solicitó información a la Seccional de Salud de Antioquia y a la Secretaría de Salud del municipio de Andes sobre todas las enfermedades de reporte obligatorio en salud, discriminando los datos solo en población masculina entre los años 2015 y 2020. El reporte otorgado por la entidad pública incluyó los eventos reportados sobre: diabetes; obesidad; intoxicación por alcohol; violencia de género e intrafamiliar; tumor maligno de próstata; infección por VIH; infección gonocócica; tumor de órganos genitales masculinos; tumor maligno de vejiga urinaria y tumor maligno de pene. Los datos reportados corresponden al número de eventos y el porcentaje de cada uno de estos, previamente enumerados en hombres mayores de edad, sin distinción social ni ningún otro parámetro de diferenciación. El análisis estadístico se realizó empleando estadística descriptiva. Conforme a la Resolución 8430 de 1993, este artículo de reflexión se clasifica como investigación sin riesgo, dado que es un estudio de análisis estadístico retrospectivo de bases de datos gubernamentales.

## RESULTADOS

En la Tabla 1 se consolida la información compartida por la Secretaría de Salud del municipio de Andes durante los años 2015 a 2020, en la que se describe la frecuencia de algunas enfermedades que afectan la salud de los hombres residentes en la mencionada localidad, que son de reporte obligatorio. Se observó que en Andes la diabetes, la obesidad y el alcoholismo son los eventos más prevalentes en los hombres. Probablemente, los datos reportados por las autoridades locales y departamentales presenten un subregistro, afirmación que puede sustentarse en que para los registros de diabetes y obesidad hay un promedio de 1 088 y 264 casos entre 2015 y 2018, en tanto que los casos bajan abruptamente en 2019 (uno y tres) y 2020 (3 y 11). Además, en reporte de intoxicación por alcohol de los años 2016, 2019 y 2020, se registran cinco, cero y seis casos, respectivamente. Lo mismo se halló en violencia intrafamiliar, pues hubo posibles subregistros en 2016, 2017 y 2019, con siete, tres y cinco casos, respectivamente. Situación similar se presenta con el cáncer de próstata, donde se encuentra que en 2016, 2018, 2019 y 2020 hubo tres, seis, tres y dos casos, respectivamente.

**Tabla 1:** Prevalencia de enfermedades que afectan la salud sexual y reproductiva en hombres de Andes, 2015-2020

Evento	n	%	n	%	n	%	n	%	n	%	n	%
Diabetes	1216	5,209	1068	4,537	1095	4,614	972	4,351	1	0,004	3	0,013
Obesidad	310	1,328	281	1,194	251	1,058	215	0,962	3	0,013	11	0,048
Intoxicación por alcohol	27	0,116	5	0,021	20	0,084	17	0,076	0	0,000	6	0,026
Violencia de género e Intrafamiliar	17	0,073	7	0,030	3	0,013	18	0,081	5	0,022	10	0,044
Tumor maligno de la próstata	20	0,086	3	0,013	21	0,088	6	0,027	3	0,013	2	0,009
Infección por VIH	13	0,056	3	0,013	9	0,038	7	0,031	8	0,035	11	0,048
Infección gonocócica	14	0,060	7	0,030	4	0,017	6	0,027	0	0,000	0	0,000
Tumor de órganos genitales masculinos	6	0,026	6	0,025	6	0,025	4	0,018	0	0,000	0	0,000
Tumor maligno de la vejiga urinaria	2	0,009	5	0,021	5	0,021	2	0,009	0	0,000	0	0,000
Tumor maligno del pene	3	0,013	2	0,008	5	0,021	1	0,004	0	0,000	0	0,000
Total	1628		1387		1419		1248		20		43	

Fuente: Secretaría de Salud de Andes, a través de registros individuales de prestación de servicios de salud y sistema de salud pública.

## DISCUSIÓN

En Colombia, la mayoría de los subprogramas sobre SSR se enfoca en prestar servicios de consejería, planificación familiar, servicios jurídicos, tratamiento de infecciones de transmisión sexual, terapia sexual y manejo de fertilidad a las mujeres, en tanto que el hombre desempeña un papel secundario y poco activo en las decisiones de sexualidad y reproducción de la pareja 3.

Con respecto a la relación entre las enfermedades más frecuentes observadas en los hombres de Andes y su efecto sobre la salud sexual, se puede afirmar que la diabetes afecta la calidad seminal porque causa hipogonadismo, mientras que la obesidad ocasiona disfunción eréctil, el alcoholismo impotencia e infertilidad, el cáncer de próstata inhibe la proliferación de células espermáticas, y la infección por VIH o por *Neisseria gonorrhoeae* puede causar uretritis, epididimitis y prostatitis 4.

La comparación de enfermedades entre la población masculina de Andes y la población masculina de la capital del departamento permitió observar que la diabetes es más prevalente en la ciudad de Medellín que en el municipio de Andes. Posiblemente, las condiciones de vida de las grandes ciudades y las influencias ambientales nocivas, además del grado de urbanización e industrialización, hacen que esta enfermedad sea más frecuente en los hombres de Medellín 5; sin embargo, no contamos con datos estadísticos que apoyen esta hipótesis. El tipo de dieta que se incorpora en la vida influye negativa o positivamente en la SSR, y por ende en la salud de la futura descendencia deseada. Así, la dieta basada en grandes proporciones de azucares, grasa saturada e ingesta de alcohol tiene consecuencias en la fertilidad de los hombres, puesto que se asocia con la resistencia a la insulina y trastornos metabólicos, como la obesidad, que inducen problemas endocrinológicos y atrofia testicular, lo que detiene muchas veces el proceso normal de espermatogénesis 4. En consecuencia, trabajos posteriores que permitan probar esta hipótesis podrían ser muy interesantes.

En los hombres de Andes, para el año 2015 se reportó una prevalencia de 0,064% de infección por *N. gonorrhoeae,* mucho menor que la reportada en otras poblaciones como Yopal, donde en una muestra de 909 hombres de edades entre 10 y 69 años se reportó una prevalencia de 1,64% de esta misma infección 6. Adicionalmente, en un estudio realizado en Esmeraldas (Ecuador) se reportó una prevalencia de 12,9% de VIH en hombres en 2016; ese mismo año, en Andes la prevalencia fue de 0,013% 7.

Según lo reportado por Hernández Vargas et al. 8, en Colombia hubo 1,60% de prevalencia de cáncer de próstata en 2018 en hombres de 40 a 49 años, resultados similares a los hallados por este estudio, en el que se encontraron prevalencias de cáncer de próstata de 0,08% y 0,32% en Andes y Medellín, respectivamente.

De acuerdo con el Instituto Nacional de Medicina Legal, en Colombia hubo un 23,7% y un 23,4% de casos de violencia contra el hombre en 2018 y 2019, respectivamente, cifra mucho mayor que la encontrada en Andes en los mismos años (0,081% y 0,022%). Así mismo, en 2015, 2016, 2017 y 2020 hubo una prevalencia de 13,3%, 14%, 14% y 13,5%, respectivamente 9, porcentajes relativamente altos si se los compara con los reportados por este estudio: 0,073%, 0,030%, 0,013% y 0,044%.

Entre las limitaciones de este trabajo se encuentran las dificultades en la búsqueda y obtención de los datos en las páginas web del Sistema Nacional de Vigilancia en Salud Pública (Sivigila) y de la Secretaría Seccional de Salud y Protección Social, debido a que se verificó el registro de eventos a nivel nacional y departamental y se observó que, si bien aparecían bastantes datos, a nivel municipal, no cumplían con nuestro objetivo porque estaban categorizados por semana y no por sexo. Por esto, se optó por solicitar datos directamente con funcionarios de la Seccional y la Secretaría de Salud del municipio de Andes. Consideramos pertinente compartir este tipo de limitaciones en el acceso a la información, con la finalidad de hacer un llamado a las entidades locales y territoriales a que dispongan de información epidemiológica de forma más abierta, de manera tal que permita al sector académico indagar, proponer y evaluar medidas que mejoren la calidad de vida de las diferentes poblaciones.

La diabetes y la obesidad son los principales problemas que afectan a los hombres de Andes, por lo que se requieren estrategias que mitiguen su impacto en esta población específica, como programas de educación sexual enfocados en los hombres ♥
